# The relationship between exercise motivation and exercise behavior in college students: The chain-mediated role of exercise climate and exercise self-efficacy

**DOI:** 10.3389/fpsyg.2023.1130654

**Published:** 2023-03-31

**Authors:** Yu Shun Zhao, Qi Shuai Ma, Xing Yi Li, Ke Lei Guo, Liu Chao

**Affiliations:** ^1^School of Physical Education, Huaibei Normal University, Huaibei, China; ^2^School of Physical Education and Health, Zhaoqing University, Zhaoqing, China

**Keywords:** exercise motivation, exercise behavior, exercise atmosphere, exercise self-efficacy, college students

## Abstract

**Objective:**

This study explored the relationship between college students’ physical activity motivation and exercise behavior and constructed a chain mediation model through the mediating roles of exercise climate and exercise self-efficacy.

**Methods:**

By random sampling, 1,032 college students were investigated using the *Exercise Motivation Scale, Physical Exercise Rating Scale, Exercise Climate Scale, and Exercise Self-Efficacy Scale.*

**Results:**

(1) There was a huge positive correlation between exercise motivation and exercise behavior (*r* = 0.240, *p* < 0.01), and the immediate ways of linking exercise motivation to exercise behavior were critical (*β* = 0.068, *t* = 0.040, *p* < 0.01). (2) Exercise motivation could positively predict exercise climate (*β* = 0.373, *t* = 0.061, *p* < 0.01) and exercise self-efficacy (*β* = 0.174, *t* = 0.039, *p* < 0.01), and exercise climate could emphatically foresee exercise behavior (*β* = 0.302, *t* = 0.051, *p* < 0.01). Exercise self-efficacy could foresee exercise behavior decidedly (*β* = 0.190, *t* = 0.048, *p* < 0.01). (3) Exercise climate and exercise self-efficacy play a critical intervening role between exercise motivation and exercise behavior. The intercession impact is explicitly made out of aberrant impacts created in three ways: exercise motivation → exercise climate → exercise behavior (mediating effect value: 0.113); exercise motivation → exercise self-efficacy → exercise behavior (mediating effect value: 0.033); exercise motivation → exercise climate → exercise self-efficacy → exercise behavior (mediating effect value: 0.027).

**Conclusion:**

(1) Exercise climate, exercise self-efficacy, and exercise behavior can all be significantly predicted by exercise motivation, suggesting that exercise motivation may help to enhance these variables. (2) In addition to having a direct impact on exercise behavior, exercise motivation can also have an indirect impact through the separate mediating effects of exercise climate and exercise self-efficacy as well as the chain mediating effect of exercise climate and exercise self-efficacy, which is crucial for encouraging college students to engage in physical activity.

## Introduction

A national strategy to advance the creation of a healthy China and enhance population health is outlined in the Healthy China 2030 Plan. It is a major measure to protect people’s health. College students will be the main force in building China in the coming decades, and their health will be widely concerned by the outside world. The eighth national survey on students’ physical health was conducted, and the results were published by the Ministry of Education in September 2021: from 2014 to 2019, the physical health of all college, middle school, and primary school students has significantly improved. Among them, the excellent and very good rate of junior high school students increased significantly by 5.1%, that of high school students by 1.8%, and that of college students by only 0.2 percentage points, indicating virtually no growth (Department of Physical Health and Arts Education Ministry of Education, 2021). A large number of studies have found that it has become an indisputable fact that the physical fitness of contemporary college students has been declining year by year. The most direct reason for the physical fitness decline of college students is the general lack of physical exercise (Yu et al., 2021). Exercise behavior refers to physical activities with a certain intensity, frequency, and duration that are carried out in leisure time with the main purpose of promoting individual health (Yan et al., 2020). Studies have found that moderate physical exercise plays a positive role in promoting human health development, such as reducing the risk of chronic diseases and improving individual mood (Jiang et al., 2017; Meng and Shen, 2018). However, college students participate in physical exercise activities with stages and instability; exercise behavior will increase when there is a physical fitness test task and stop when there is no physical fitness test task. During the period of heavy learning tasks and final exams, exercise will be stopped. After this period, exercise behavior will increase again (Tian et al., 2018). A study of university postgraduates found that their health condition was worse than that of undergraduates. The reason was that postgraduates did not participate in physical exercise seriously enough, with fewer times, shorter times, and less intensity (Liu, 2020b). The physical health of college students has continued to deteriorate.

### Exercise motivation and exercise behavior

The term “motivation” refers to the psychological or emotional force that propels a person to engage in certain behaviors, while exercise motivation is the internal driving force and psychological motivation that drives an individual to engage in physical exercise, which determines the purpose, intensity, frequency, and effect of an individual’s participation in physical exercise and is the direct cause of exercise behavior (Xu, 2020). Self-Determination Theory (SDT) holds that individual behavior can be divided into self-determined behavior and non-self-determined behavior, and the two behaviors are driven by three modes of motivation. Self-determined behaviors are driven by internal motivations, while non-self-determined behaviors are driven by external and unmotivated motivations (Jacobs, 2000). This theory holds that when individual exercise behavior is driven by internal motivation, it is conducive to the formation of good exercise habits. Liu (2010) found that college students’ exercise motivation was related to exercise time, exercise intensity, and exercise behavior to some extent. In this manner, there was a huge relationship between exercise motivation and exercise behavior. Wei et al. (2020) discovered in a review of the elderly that the fulfillment of basic mental necessities could well predict the exercise behavior of the elderly *via* the intermediary factor of self-assurance and inspiration. Besides, investigations have discovered that exercise motivation straightforwardly advances and decidedly predicts exercise behavior (Yang et al., 2015). In light of this, hypothesis 1 is advanced: exercise motivation can definitely predict exercise behavior.

### The mediating role of exercise climate

According to SDT and the hierarchy of motivation theory, the process of internalizing individual motivation is influenced by external environmental factors, so climate may play an important role in exercise motivation and exercise behavior. The achievement goal theory suggests that an individual’s motivation is derived from goal orientation or task orientation, so scholar Ames has introduced the concept of “motivational climate” as an antecedent variable for internalizing motivation based on the achievement goal theory. There is also a common climate called “exercise climate,” and the concept of exercise climate refers to the exercise environment created by the surrounding people and the exercise information available to the exercise participants. Both motivational and exercise climates are different kinds of climates, and exercise climates, as typical representatives of external environmental factors, have an important impact on university students’ physical activity (Wang, 2017). Studies have proven that the exercise environment is one of the most important factors in encouraging college students to actively and independently engage in physical exercise activities (Hu et al., 2020). A good exercise climate is of great significance for improving college students’ exercise behaviors. A natural atmosphere, such as a good field and first-class equipment during exercise, can attract college students to frequent physical exercise, stimulate their interest and enthusiasm to participate in physical exercise, and gradually guide and increase their exercise behavior (Yang, 2016). Therefore, exercise climate may play an important role in predicting college students’ exercise behavior.

According to research, physical exercise motivation has an effect on the exercise environment, which may have a predictive effect (Hu and Wei, 2005). Li and Yang (2016) investigated square dancing groups and found that both motivation and exercise climate had an impact on exercise persistence. At the same time, physical exercise motivation influences the exercise environment. Therefore, exercise motivation may play an important role in predicting the exercise climate. In conclusion, exercise motivation may be closely related to exercise climate, and exercise behavior can be further predicted by exercise climate. As a result, it is suggested that exercise climate acts as a mediator between exercise motivation and exercise behavior in this study’s second hypothesis.

### The mediating role of exercise self-efficacy

Exercise self-efficacy may likewise assume a significant role in the connection between exercise motivation and exercise behavior. As indicated by the Environmental Model of Active Work (EMAW), mental variables are viewed as another significant component influencing exercise behavior. Exercise self-efficacy is the mental element variable most firmly connected with exercise behavior (Sallis et al., 1992). Exercise self-efficacy alludes to the mental capacity of a person to accept that they can finish the laid-out objectives and errands of physical exercise. It is an important concept in the cognitive theory of exercise and can directly affect the level of exercise behavior. Exercise self-efficacy can regulate personal emotions in physical exercise so that individuals can reasonably control negative emotions and deal with problems with a positive attitude when faced with difficulties in exercise (Dong et al., 2018). Investigations have discovered that the more grounded the exercise self-efficacy of people, the more effectively they are taking part in practice ways of behaving, and the recurrence, force, and season of activity will expand (Guo et al., 2010). Likewise, a review demonstrates the way that dynamic cooperation in actual activity can precisely predict the self-efficacy of center school understudies and cause them to keep a hopeful and bright mind-set, producing drive in exercise behavior (Yuan and Zhang, 2015). Subsequently, exercise self-efficacy might assume a significant role in predicting the exercise behavior of undergraduates.

The exploration observed that there is a critical positive connection between exercise motivation and self-efficacy, and exercise motivation can definitely influence the certainty of undergrads who take part in working out (Cheng and Xu, 2016). Dong et al. (2020) found that exercise self-efficacy meaningfully affected teenagers’ cooperation in exercising. Generally speaking, individuals with high exercise motivation will have a better sense of exercise self-efficacy and thus form more active exercise behaviors. The reason is that people with strong exercise motivation often dare to face difficulties and setbacks in physical exercise and show a strong sense of self-efficacy. Those who are less motivated to exercise will avoid challenges. Therefore, exercise motivation may be an important predictor of exercise self-efficacy. In conclusion, exercise motivation may be closely related to exercise self-efficacy, and exercise self-efficacy can further predict exercise behavior. Based on this, hypothesis 3 of this study is proposed: exercise self-efficacy plays a mediating role between exercise motivation and exercise behavior.

### The chain mediated role of exercise climate and exercise self-efficacy

The behavioral ecological model of exercise puts forward the theory that environmental factors, physiological factors, and psychological factors will interact with each other and jointly affect individual exercise behavior (Yang et al., 2014). As an external factor of the environment, the exercise climate provides a decision-making basis and driving effect for individuals to decide whether to do physical exercise. Exercise self-efficacy, as an internal factor of psychological factors, provides support for whether individuals choose physical exercise or whether they can persist when encountering difficulties in physical exercise. Dong et al. (2022) found that the interpersonal circle of individuals who share the same interest in exercise will gradually form a good exercise climate, making individuals feel the pleasure brought by exercise and thus improving the self-efficacy of exercise. Different examinations have found that the exercise climate meaningfully affects understudies’ exercise self-efficacy, and the relational association, normal association, and data obtained in the exercise climate all assume a significant part in understudies’ exercise self-efficacy. Undergrads practice in a decent exercise climate; they practice together, empower, progress, and become together; continuously, actual activity will create serious areas of strength for a person to work out, extraordinarily preparing the excitement of understudies to partake in actual activity. Exercise will likewise build your identity’s viability. Hence, hypothesis 4 of this study is proposed: exercise climate and exercise self-efficacy play an intervening role between exercise motivation and exercise behavior.

**Figure 1:**
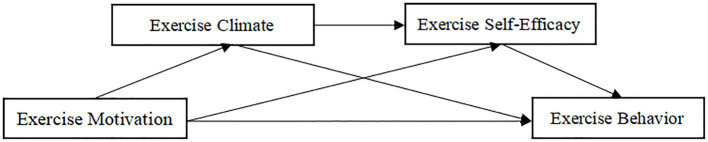
Hypothetical model.

To summarize, to examine the interior component between exercise motivation and exercise behavior, this study means to fabricate a chain intervention model (as displayed in Figure 1) and check the accompanying perspectives: (1) exercise motivation fundamentally and emphatically predicts exercise behavior of understudies; (2) exercise climate plays a free interceding job between exercise motivation and exercise behavior of understudies; (3) exercise self-efficacy plays an autonomous intervening job between exercise motivation and exercise behavior of understudies; and (4) exercise climate and exercise self-efficacy play a chain interceding job between exercise motivation and exercise behavior of understudies.

## Materials and methods

### Procedure and participants

A random sampling method was adopted to conduct a questionnaire survey among students from six colleges and universities in Anhui Province. Due to the impact of the epidemic, this survey used the platform of the Juanxing Network to issue electronic questionnaires. The purpose of the research is explained to the respondents in the questionnaire guidance, and they promise to keep the content they fill in strictly confidential. The questionnaire includes five parts: basic information filling, an exercise motivation scale, an exercise grade scale, an exercise climate scale, and an exercise self-efficacy scale. A total of 1,088 questionnaires were collected; 56 were excluded from regular answers and invalid questionnaires, and 1,032 were valid, with a recovery rate of 94.85%. Among them, 530 were boys and 502 were girls.

### Measures and instruments

#### Exercise motivation

College students’ exercise motivation was measured using the *Exercise Motivation Scale* (MAM-R) simplified version, which Chen et al. (2013) amended. Research has shown that the scale is appropriate for assessing Chinese college students’ exercise motivation (Li and Li, 2020). The scale has 15 elements altogether and is broken down into five categories: fun motivation, ability motivation, appearance motivation, health motivation, and social motivation (e.g., “I want to have a strong body”). The Likert scale employs five points, from “strongly disagree” to “strongly agree,” for each response. The individuals were more inclined to exercise if their ratings were higher. The three exercise motivation scale items in this study converge on one factor with a KMO value of 0.93 and a Chi-square worth of 5654.78 (*p* < 0.01), representing 64.39% of the all-out difference (Table 1). The fitting file of the corroborative element examination was *χ^2^/df = 4.082, CFI = 0.965, NFI = 0.954, GFI = 0.939, TLI = 0.942, and RMESA = 0.077*, and the decency of fit was fundamentally better, demonstrating that the scale had great primary legitimacy (Table 2). In this review, *Cronbach’s α* was 0.95, showing great interior consistency of the scale.

**Table 1:** Exploratory factor analysis and internal consistency test results.

Factor naming	KMO	Bartlett chi square value (*p*-value)	Cumulative variance interpretation rate	Cronbach’ coefficient
EM	0.93	5654.78 (*p* < 0.01)	64.39%	0.95
EC	0.86	6105.34 (*p* < 0.01)	77.21%	0.76
ESE	0.93	4051.30 (*p* < 0.01)	58.79%	0.94
EB	0.68	411.17 (*p* < 0.01)	68.44%	0.77

EM, exercise motivation; EC, exercise climate; ESE, exercise self-efficacy; EB, exercise behavior.

**Table 2:** Confirmatory factor analysis results.

Factor naming	χ^2^/df	CFI	NFI	GFI	TLI	RMESA
EM	4.082	0.965	0.954	0.939	0.942	0.077
EC	4.167	0.965	0.955	0.940	0.929	0.078
ESE	3.748	0.972	0.962	0.954	0.955	0.073
EB	3.792	0.932	0.911	0.917	0.925	0.093

EM, exercise motivation; EC, exercise climate; ESE, exercise self-efficacy; EB, exercise behavior.

#### Exercise climate

*The Exercise Climate Scale* was adapted from the Liu et al. (2011) outdoor exercise climate scale for teenagers, which has five components and 17 items in total. The scale uses Likert 5 points, from “not at all” to “extremely strong,” on a scale from 0 to 1. The workout atmosphere is felt more strongly the higher the score. Physical activity is substituted for key terms like “sports” and “outdoor sports” (e.g., “During the process of taking part in physical exercise, my friendship with my partners has been deepened”). Research has shown that this scale is appropriate for assessing how Chinese college students perceive the exercise climate (Dong et al., 2022). The 17 components on the exercise climate scale in this study well converged to one factor, whose KMO esteem was 0.86, and the Chi-square worth of the Bartlett round test was 6105.34 (*p* < 0.01), representing 77.21% of the absolute difference (Table 1). The fitting file of the corroborative component examination was *χ^2^/df = 4.167, CFI = 0.965, NFI = 0.955, GFI = 0.940, TLI = 0.929, and RMESA = 0.078*, and the decency of fit was fundamentally better, showing that the scale had great underlying legitimacy (Table 2). In this review, *Cronbach’s α* was 0.76, showing great inside consistency of the scale.

#### Exercise self-efficacy

*The Exercise Self-Efficacy Scale* developed by Wu et al. (2002) is designed to assess people’s propensity for attitude following exercise-contradicting circumstances. There are 12 items on the scale (e.g., “I will keep exercising even if I feel tired”) to “I’m sure I can do it” from “I cannot do it.” The level of exercising self-efficacy increases with score. A prior study demonstrated that Chinese college students can effectively use this measure (Dong et al., 2022). The 12 items on the exercise self-efficacy measure in this study converge nicely to one factor, accounting for 58.79% of the total variance with a KMO value of 0.93 and a Chi-square value of 4051.30 (*p < 0.01*) representing 58.79% of the complete difference (Table 1). The fitting list of the corroborative component examination was *χ^2^/df = 3.748, CFI = 0.972, NFI = 0.962, GFI = 0.954, TLI = 0.955, and RMESA = 0.073*. The decency of fit was altogether better, showing that the scale had great underlying legitimacy (Table 2). In this review, *Cronbach’s α* was 0.94, demonstrating the great internal consistency of the scale.

#### Exercise behavior

To assess the quantity of physical activity from three perspectives—intensity, time, and frequency—the *Physical Activity Rating Scale* (PARS-3) developed by Liang (1994) was used. Physical activity scores range from 0 to 100, with 100 being the highest possible score. The more points you receive, the more exercise you receive. For low exercise, the total score is 19 or less; for moderate exercise, it ranges from 20 to 42; and for vigorous exercise, it ranges from 43. This study divided the quantity of light physical activity into two categories: no physical activity and light exercise. No physical activity is equal to or less than 4 points, and a small workout ranges from 5 to 19. As a result, there were four categories of physical activity in this study, ranging from “no physical activity” to “a great deal of physical activity.” A prior study demonstrated that Chinese college students can effectively use this measure (Liu, 2020a). With a KMO value of 0.68 and a Chi-square value of 411.17 (*p* < 0.01), the three physical activity rating scale items in this study converge nicely to one factor, accounting for 68.44% of the total variance (Table 1). The scale exhibited strong structural validity, as evidenced by the confirmatory factor analysis’s fitting index, which was *χ^2^/df = 3.792, CFI = 0.932, NFI = 0.911, GFI = 0.917, TLI = 0.925, and RMESA = 0.093* (Table 2). *Cronbach’s α* in this study was 0.77, indicating that the scale had acceptable internal consistency.

#### Processing of data

In this review, IBM SPSS 26.0 and AMOS 21.0 factual programming were utilized for all information examination. After the poll was gathered, all information was handled as follows: (1) Exploratory variable investigation was led for all scales utilizing SPSS 26.0; (2) Corroborative component examination was performed for all scales utilizing AMOS 21.0; (3) SPSS 26.0 was utilized to test the inner consistency of all scales; (4) “Harman single element strategy” was utilized for the normal technique deviation test; and (5) Pearson connection investigation with SPSS 26.0 was utilized to compute the connection between exercise motivation, exercise climate, exercise self-efficacy, and exercise behavior. The mean (M) and standard deviation (SD) of persistent factors with a typical dissemination were utilized. (6) The SPSS full-scale program ordered by Hayes in SPSS 26.0 was utilized to confirm the intervening job of exercise climate and exercise self-efficacy in the connection between exercise motivation and exercise behavior and the chain intervening job of exercise climate and exercise self-efficacy in the connection between exercise motivation and exercise behavior; (7) Model 6 in the SPSS full-scale program aggregated by Hayes was utilized for the chain intervention test. In this review, the significance level was set at *p* < 0.05.

## Results

### Common method bias test

The term “normal technique bias” refers to the erroneous covariation among indicator and rule factors caused by a similar information source or rater, a similar estimation climate, the venture setting, and the characteristics of the actual project. This fake correlation, which truly confounds the consequences of the review and possibly deceives the determinations, is a deliberate blunder. Since the information in this study was all gathered from surveys completed by subjects with emotional goals, there were a few strategic blunders. To stay away from such predispositions, the Harman single component examination was utilized to confirm whether the survey had explicit, normal systemic inclinations. An exploratory element investigation was performed on each of the 47 estimated scale questions. The outcomes showed that among the 8 elements with an eigenvalue more prominent than 1, the difference in translation pace of the primary variable was 29.68%, which was lower than the basic record of 40%. Subsequently, there was no normal technique predisposition in this study’s information.

### Descriptive statistics and correlation analysis of variables

Table 3 presents the mean value, standard deviation, and correlation coefficient of variables in detail. It can be seen from the data in Table 3 that exercise motivation (*r* = 0.240, *p* < 0.01), exercise climate (*r* = 0.412, *p* < 0.01), and exercise self-efficacy (*r* = 0.336, *p* < 0.01) are significantly positively correlated with exercise behavior. Exercise motivation (*r* = 0.316, *p* < 0.01) and exercise climate (*r* = 0.444, *p* < 0.01) were positively correlated with exercise self-efficacy. Exercise motivation (*r* = 0.373, *p* < 0.01) was found to be positively related to exercise environment. The correlation analysis results provide preliminary support for the subsequent hypothesis testing.

**Table 3:** Means, standard deviations, and correlations among variables.

Variable	*M*	*SD*	1	2	3	4
1. EM	4.53	0.55	1			
2. EC	3.72	0.52	0.373**	1		
3. ESE	2.11	0.54	0.316**	0.444**	1	
4. EB	33.18	27.55	0.240**	0.412**	0.336**	1

*N* = 1,032. EM, exercise motivation; EC, exercise climate; ESE, exercise self-efficacy; EB, exercise behavior, **p < 0.01.

### The mediation effect test between exercise climate and exercise self-efficacy

As per Wen Zhonglin and Ye Baojuan’s idea on the intervention impact test, the chain intercession impact model is tested (Wen and Ye, 2014), and the experimental outcomes are displayed in Table 4. As per the information in Table 4, exercise motivation can fundamentally and emphatically predict the exercise behavior of understudies, with a complete impact of 0.240 (*p* < 0.01) and an immediate impact of 0.068 (*p* < 0.01). In this manner, hypothesis 1 is substantial. At the point when exercise climate and exercise self-efficacy were remembered for the relapse condition, exercise motivation decidedly anticipated exercise climate (*β* = 0.373, *p* < 0.01) and exercise motivation (*β* = 0.174, *p* < 0.01). Exercise climate anticipated exercise self-efficacy (*β* = 0.380, *p* < 0.01) and exercise behavior (*β* = 0.302, *p* < 0.01). Exercise self-efficacy anticipated exercise behavior emphatically (*β* = 0.190, *p* < 0.01). Simultaneously, exercise motivation might in any case anticipate exercise behavior emphatically (*β* = 0.068, *p* < 0.01). It very well may be presumed that exercise climate and exercise self-efficacy play incompletely interfering roles between exercise motivation and exercise behavior, separately. Hypothesis 2 and 3 are upheld and confirmed by information.

**Table 4:** Analysis of regression relationship among variables.

Effect	Item	Effect	SE	*t*	*p*	LLCI	ULCI
Direct effect	EM ⇒ EB	0.068	0.040	1.705	<0.01	0.010	0.145
Indirect effect	EM ⇒ EC	0.373	0.061	6.128	<0.01	0.253	0.492
	EM ⇒ ESE	0.174	0.039	4.524	<0.01	0.099	0.250
	EC ⇒ ESE	0.380	0.037	10.352	<0.01	0.308	0.452
	EC ⇒ EB	0.302	0.051	5.958	<0.01	0.203	0.402
	ESE ⇒ EB	0.190	0.048	3.995	<0.01	0.097	0.284
Total effect	EM ⇒ EB	0.240	0.048	5.047	<0.01	0.147	0.334

EM, exercise motivation; EC, exercise climate; ESE, exercise self-efficacy; EB, exercise behavior.

**Figure 2:**
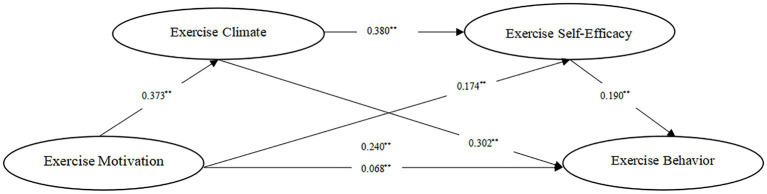
Chain mediation model. **p < 0.01.

The Bootstrap intercession test technique (Cole et al., 2008) was utilized to further test the intercession impact, and the Cycle module was utilized to fabricate an underlying condition model. Model 6 was chosen from the Cycle module to test the chain intervention impact of exercise climate and exercise self-efficacy. The experimental outcomes are displayed in Table 5. The certainty timespan trial of the intervening impact between exercise climate, exercise motivation, and exercise behavior is (0.069, 0.173), barring 0, and the interceding impact is tried. Hypothesis 2 is substantial, and its intervening impact represents 47.08% of the absolute impact. The certainty time period intervening in the impact trial of exercise self-efficacy between exercise motivation and exercise behavior is (0.017, 0.057), barring 0. The intervening impact has been tried. Hypothesis 3 is legitimate, and its intervening impact represents 13.75% of the complete impact, is not exactly the intervening impact of the exercise climate. The certainty timespan chain intervening impact way between exercise climate and exercise self-efficacy does exclude the number 0 (0.013, 0.044), and the chain intervening impact way exists, representing 11.25%. Hypothesis 4 is valid. The intervening impact of exercise climate and exercise self-efficacy on exercise motivation and exercise behavior is displayed in Figure 2.

**Table 5:** Mediating effect and effect size.

Path	Effect	Boot SE	Boot LLCI	Boot ULCI
EM ⇒ EC ⇒ EB	0.113	0.027	0.069	0.173
EM ⇒ ESE ⇒ EB	0.033	0.010	0.017	0.057
EM ⇒ EC ⇒ ESE ⇒ EB	0.027	0.008	0.013	0.044

EM, exercise motivation; EC, exercise climate; ESE, exercise self-efficacy; EB, exercise behavior.

## Discussion

In this study, a chain mediation effect model was constructed to comprehensively explore the influence of exercise motivation, exercise climate, and exercise self-efficacy on exercise behavior and mainly explore the influence mechanisms of exercise climate and exercise self-efficacy on exercise motivation and exercise behavior, which is helpful to fully reveal the influence mechanism of exercise behavior. The results of this study have certain theoretical and practical significations for encouraging college students to develop good physical exercise habits and enhance their physical fitness.

### Exercise motivation and exercise behavior

According to the findings of earlier pertinent studies, there is a considerable positive link between exercise habits and motivation for exercise (Xu, 2021) and verifies hypothesis 1. Xu (2021) believes that exercise motivation will promote exercise behavior, which further confirms that people with high exercise motivation will have more psychological needs for physical exercise and thus increase exercise behavior. The motivation for physical exercise has five dimensions, which are fun motivation, ability motivation, appearance motivation, health motivation, and social motivation. The fun motivation is to experience the pleasure brought by sports in physical exercise, which will make them physically and mentally happy. Competency motivation is the desire to learn new motor skills or improve the original motor skills through physical exercise. The appearance motivation is the need to get fit through physical exercise; the health motivation is to strengthen the body through physical exercise so that they have a healthy physique; and the social motivation is social motivation. I want to make friends and expand my circle of friends through physical exercise. According to the actual research results of various scholars, exercise motivation is often different in different genders and ages, and the motivation to participate in exercise behavior is diverse (Huang and Qiu, 2019). Studies have shown that most students take physical and mental health promotion as their main motivation (Zhang and Zhao, 2009). Girls’ motivation for physical exercise is mainly physical appearance, while boys pay more attention to ability motivation and social motivation (Kilpatrick et al., 2005). According to Zhu (2019), college students’ motivation and exercise activity are correlated, although there is a gender difference, with boys often showing higher levels of motivation. It was also mentioned that college students’ exercise behavior increased when they had a considerable amount of motivation to exercise. Wei (2010) found in his research that freshmen have a strong motivation for physical exercise. The reason is that freshmen have just entered the school and are full of curiosity and desire for the new environment. Therefore, they set many goals and are determined to complete them, which enhances their exercise motivation. Sophomores and juniors have been living on campus for a while, and students have gradually found their interests on campus. Physical exercise may be a small part of them, so the motivation for physical exercise will decrease. Senior students gradually realize the importance of physical exercise. Therefore, the exercise motivation will improve, but due to the pressure of an internship, writing papers, and so on, the time and frequency of actual exercise will be limited. Others are motivated by the occurrence of specific events. For example, in the study of Yuan and You (2021), it was found that, against the background of the COVID-19 epidemic, the motivation of college students to exercise and fight against the epidemic was also one of the reasons for them to do physical exercise. In general, exercise motivation is the primary factor that determines individual exercise behavior.

### The independent mediating effect of exercise climate

In this study, it was found that exercise climate played a mediating role between exercise motivation and exercise behavior, and hypothesis 2 was verified. This is consistent with previous research evidence, namely, that exercise motivation significantly positively predicts exercise climate (Hu and Wei, 2005), and exercise climate significantly positively predicts exercise behavior (Jiang et al., 2004). In this study, the three variables were investigated at the same time, revealing that exercise motivation is an important factor to improve the exercise climate and improve exercise behavior.

The Ecological Model of Physical Activity (EMPA) suggests that the formation and change of physical activity behavior must be examined in relation to external environmental factors (John and Rebecca, 2003). Exercise climate often plays an important role as an external environmental factor that enhances individual exercise behavior. One side, exercise motivation has a positive predictive effect on the exercise climate. Exercise motivation has a unique influence on the exercise climate, and both motivation and atmosphere are factors that influence individuals’ adherence to physical exercise (Liu et al., 2005). College campuses provide good sports venues and sports equipment for college students, attract more and more students to do physical exercise, and gradually form a positive exercise climate. A decent exercise climate can invigorate the excitement of undergrads to partake in actual activity and increment their time, power, and recurrence of actual activity. The expansion in actual activity is helpful for the advancement of the physical and psychological wellness of undergrads and the improvement of coordinated movements and social capacity.

Then again, the exercise climate emphatically anticipated exercise behavior. The exercise climate essentially affects whether contemporary understudies participate in actual activity; it straightforwardly decides the understudies’ sports awareness and ideas and fills in as an exogenous power to urge understudies to participate in actual activity. A decent exercise climate can straightforwardly direct undergraduates to partake in sports exercises and act as an illustration in the sport activity. But the poor exercise climate will hinder the pace of physical exercise by college students, reduce their enthusiasm to participate in sports, restrain their desire to exercise, and even make them interrupt or quit physical exercise. In addition, according to the theory of “three factors of emotion” proposed by Wendell, emotion is produced by the interaction of external stimuli, physiological changes in the body, and cognitive processes. When the exercise environment around the individual is stronger, the cognition level of physical exercise rises, and the individual gradually produces an exercise mood, which encourages college students to adhere to physical exercise more closely. Therefore, on the premise of providing exercise places for students, colleges and universities should often organize sports events, create a good exercise climate, guide college students to participate in physical activity, enhance their exercise motivation, and stimulate their interest in physical exercise to increase the frequency, intensity, and time of exercise behavior.

### The independent mediating effect of exercise self-efficacy

This investigation discovered that exercise self-efficacy played an intervening role between exercise motivation and exercise behavior, which affirmed theory 3. This is consistent with past examination proof, specifically that exercise motivation essentially emphatically predicts exercise self-efficacy (Dong et al., 2020) and exercise self-efficacy fundamentally decides exercise behavior (Dong et al., 2022). In this review, the three factors were researched simultaneously, uncovering that exercise motivation is a significant element to work on the feeling of exercise self-efficacy as well as a significant variable to increase exercise behavior.

According to the health behavior process model, high self-efficacy is an influential factor between exercise motivation and exercise behavior. Exercise motivation can positively predict exercise self-efficacy. College students with strong exercise motivation dare to face and overcome the difficulties and setbacks of participating in physical exercise, showing a strong sense of exercise self-efficacy. However, college students with weak exercise motivation will also have a low sense of self-efficacy in exercise. When they encounter difficulties, they will choose to avoid them rather than try to solve them, which will lead to the interruption or even termination of their exercise behavior.

Meanwhile, exercise self-efficacy can also affect exercise behavior. Exercise self-efficacy is the level of confidence that people have in their athletic ability. The easier it is to enjoy physical activity, increase the durability of exercise behavior, firmly exercise all the time, and form a lifelong habit of sports, the higher the exercise self-efficacy. Therefore, college students should gradually form the concept of regular physical exercise. Nowadays, it is very important to have a healthy body, and physical exercise is the best prescription. Physical activity can improve self-efficacy, improve mood, and have a positive impact on lifelong development.

### The chain mediated effect of exercise climate and exercise self-efficacy

In view of the prior, a chain intercession model was created to research the cycle and system of exercise motivation that advances exercise behavior. Exercise climate and exercise self-efficacy play various intervening roles between exercise motivation and exercise behavior. Great exercise motivation, specifically, can further develop the activity climate around people, and exercise climate is a significant factor working on the improvement of exercise self-efficacy in people, and exercise self-efficacy will additionally influence people’s exercise behavior. This affirms speculation 4 of this review. A few investigations have discovered that exercise climate and exercise self-efficacy play an intervening role between exercise motivation and exercise behavior (Dong et al., 2022). It is brought up that the exercise climate meaningfully affects undergrads’ self-efficacy in working out. The stronger the exercise climate around college students is, the deeper their perception of physical exercise will be, which is conducive to forming positive emotions and promoting the improvement of college students’ self-efficacy in exercise. Therefore, a good exercise climate can improve individual self-efficacy in exercise, stimulate college students’ motivation to exercise, and increase exercise behavior.

Subsequently, the chain intercession of exercise climate and exercise self-efficacy in this study is doable, and it can have an impact on the interceding impact of exercise motivation on exercise behavior. In this way, involving exercise climate and exercise self-efficacy as the “third factor” to overcome any barrier between exercise motivation and exercise behavior is useful to make sense of and anticipate the perplexing component of the change from motivation to behavior and has a specific directing incentive for advancing exercise behavior in undergraduates.

### Practical significance

This study looks at the impact of exercise motivation on exercise behavior, advances pertinent examination in the field of exercise motivation and exercise behavior, and has specific reasonable importance for further developing the exercise behavior of understudies. Firstly, exercise motivation is an important predictor of exercise behavior. The physical fitness of college students is declining year by year, and they lack serious physical exercise, which should be given full attention. Exercise behavior is related to exercise environment and exercise self-efficacy. Exercise motivation can not only positively predict exercise climate and exercise self-efficacy but also play an important role in predicting the exercise behavior of college students. Therefore, improving the motivation of college students’ physical exercise should become an important part of a college education. According to the characteristics of the physical and mental development of students, physical education teachers should create conditions for students to do good physical education and extracurricular physical exercise, which can provide direct help to stimulate the exercise motivation of college students. Secondly, exercise climate and exercise self-efficacy are important factors affecting college students’ exercise behavior. The chained impact of exercise climate and exercise self-efficacy suggests that sports instructors should focus on the exercise behavior of understudies because of exercise climate and exercise self-efficacy. Carry out the difference in understudies from “propelled” to “activity,” advance the exercise behavior of undergrads, upgrade the exercise motivation simultaneously, and focus on upgrading the exercise climate around undergrads and working the fair and square of exercise self-efficacy to expand the exercise behavior of undergrads.

### Limitations and prospects

Most importantly, this paper takes on an emotional detailing strategy to quantify understudies’ exercise behavior, which is heavily impacted by individual subjectivity, so the causal connection between factors cannot be surmised. Later on, longitudinal following or an exploratory mediation configuration can be utilized to make even more sense of the impact of exercise motivation on the exercise behavior of understudies. Thirdly, the choice of subjects from a similar region is helpful for working on the interior legitimacy of the review, but it restricts the outside legitimacy of the review. Future examinations can zero in on various kinds of colleges in various territories for examination and exploration. At last, this concentration just considers the interceding impact of exercise climate and exercise self-efficacy on exercise motivation and exercise behavior of understudies, yet actually, there might be other intervening factors, for example, peer support, social help, and instructors’ ability to instruct, that need further exploration.

## Conclusion

This paper investigates the huge intervening impact of exercise climate and exercise self-efficacy on exercise motivation and behavior. What’s more, there are three explicit interceding ways: (1) the single intervening impact of exercise climate; (2) the sole intervening impact of exercise self-efficacy; and (3) the chain interceding impact of exercise climate and exercise self-efficacy. Through the inside and outside investigation of the survey information of undergrads, the outcomes show that exercise motivation, exercise climate, exercise self-efficacy, and exercise behavior are altogether associated, and exercise motivation can essentially predict exercise behavior. It very well may be found in this paper that undergrads’ exercise behavior is impacted by the encompassing exercise climate and the degree of exercise self-efficacy. Subsequently, during the time spent further developing understudies’ exercise behaviors, more consideration ought to be paid to the exercise climate around understudies as well as individual self-efficacy of exercise.

## Data availability statement

The original contributions presented in the study are included in the article/supplementary material, further inquiries can be directed to the corresponding authors.

## Ethics statement

Ethical review and approval was not required for the study on human participants in accordance with the local legislation and institutional requirements. Written informed consent from the patients/ participants was not required to participate in this study in accordance with the national legislation and the institutional requirements.

## Author contributions

YZ and KG designed the study, collected and analyzed the data, and wrote the manuscript. QM, XL, and LC revised the manuscript. All authors contributed to the article and approved the submitted version.

## Conflict of interest

The authors declare that the research was conducted in the absence of any commercial or financial relationships that could be construed as a potential conflict of interest.

## Publisher’s note

All claims expressed in this article are solely those of the authors and do not necessarily represent those of their affiliated organizations, or those of the publisher, the editors and the reviewers. Any product that may be evaluated in this article, or claim that may be made by its manufacturer, is not guaranteed or endorsed by the publisher.
